# Repair of large segmental bone defects with fascial flap-wrapped allogeneic bone

**DOI:** 10.1186/s13018-016-0492-9

**Published:** 2016-12-15

**Authors:** Honglei Dou, Guowei Wang, Na Xing, Lina Zhang

**Affiliations:** 1 Department of Orthopaedics, Yidu Central Hospital Affiliated to Weifang Medical University, Weifang, 261000 Shandong Province China; 2 Department of Anesthesiology, Qingdao Municipal Hospital, Qingdao, 266071 Shandong Province China

**Keywords:** Fascial flap, Allogeneic bone, Bone defects

## Abstract

**Background:**

The objective of this study was to investigate the therapeutic effects of the application of fascial flap-wrapped allogeneic bone for repair of large segmental tibial defects in rabbits and provide a theoretical basis for treatment of large segmental defects in weight-bearing bones.

**Methods:**

Forty-eight healthy adult New Zealand White (NZW) rabbits were randomized into two groups to establish 15-mm bone defects in the proximal tibia. Bone defects in test and control groups were repaired using allogeneic bone with and without a vascularized fascial flap from the rabbit proximal tibia, respectively. The differences in repair of bone defects between the two groups were assessed with postoperative X-ray examination, new bone quantity assessment, serum bone Gla protein (BGP) level, and biomechanical testing.

**Results:**

The therapeutic effect in the test group was superior to that in the control group.

**Conclusions:**

Fascial flap-wrapped allogeneic bone is superior to allogeneic bone alone, and is ideal for the treatment of large segmental bone defects.

## Background

Large segmental bone defects with various causes are common and difficult to reconstruct. Treatments of bone defects mainly include grafting with autogenous free bone, vascularized pedicled bone, bone with microvascular anastomosis, and allogeneic, xenogeneic, and synthetic bone. Large segmental defects in weight-bearing bone (>6 cm) are difficult to reconstruct with autogenous free bone grafts because of the lack of blood supply, long process of revascularization, and creeping substitution [1], or with allogeneic bone grafts because of immunological rejection [2]. Therefore, repair of bone defects has focused on vascularized bone grafts from the iliac crest, scapula, rib, and fibula. These methods have sure effects for repair of small segmental tibial defects, but for large segmental defects in weight-bearing bones, the deficiencies such as insufficient grafting bone strength, long external fixation time, vascular anastomosis mismatch, and insufficient bone supply still exist [3–10]. Therefore, fascial flap-wrapped allogeneic bones with vascular pedicles have been widely used to repair bone defects due to advantages such as abundance of sources, various shapes and sizes, good bioactivity, and biocompatibility.

## Methods

### Animals and grouping

Forty-eight 6-month-old healthy New Zealand White rabbits (3.0–3.5 kg) were evenly randomized into two groups. The bone defect model was established. Bone defects in test (A) and control (B) groups were repaired using allogeneic bone with and without vascularized fascial flaps from the rabbit proximal tibia, respectively. This study was carried out in strict accordance with the recommendations in the Guide for the Care and Use of Laboratory Animals of the National Institutes of Health. The animal use protocol has been reviewed and approved by the Institutional Animal Care and Use Committee (IACUC) of Weifang Medical University.

### Establishment of bone defect model and repair methods

**Fig. 1:**
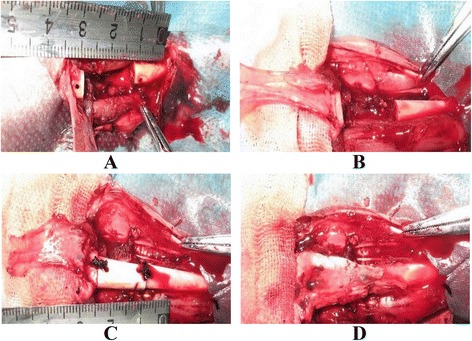
Surgery steps. **a** Standard large segmental bone defect was prepared. **b** Fascial flap was cut. **c** Allogeneic bone was implanted and fixed. **d** Allogeneic bone was wrapped by fascial flap

Rabbits were anesthetized by intraperitoneal injection with 50 mg/kg ketamine (Fujian Gutian Pharma., Co., Ltd., Fuzhou, China) and fixed in the supine position. After routine disinfection and draping, a 3-cm incision was made on the anteromedial aspect of the tibia, the skin and subcutaneous tissue were cut, and the pretibial muscles were pushed aside without damaging anterior tibial vessels or their branches. In the test group, the vascularized fascial flap with proximal vascular pedicle was excised from any site around the tibia. To establish a standard large segmental bone defect model, 15 mm of tibial shaft with periosteum was excised below the tibial tubercle. The fibula was kept intact, and a 1.0-mm Kirschner wire was used to make a hole at both the distal and proximal ends of the defect site. Cryopreserved allogeneic bone was inserted into the defect site, tied with silk thread using the holes, and wrapped with a fascial flap. The tissue layers were sutured and the wound was dressed. In the control group, a bone defect was established using the same method without wrapping the allogeneic bone with a fascial flap. Finally, plaster fixation was performed on the rabbits in both groups. Each rabbit was fed in a single cage, and received intramuscular gentamicin for 3 days after surgery (Fig. 1). No infection occurred in all rabbits. At 2, 4, 8, 12, and 16 weeks after surgery, 4, 4, 4, 8, and 4 rabbits in each group, respectively, were sacrificed with an air embolism method for further examination.

### X-ray examination

Four, 8, 12, and 16 weeks after surgery, X-rays were taken of the repaired side in four rabbits in each group to inspect bone callus formation and bone healing, and were evaluated by the Lane-Sandhu Scoring System [11].

### New bone quantity assessment by optical microscopy

Two, 4, 8, and 12 weeks after surgery, tibial specimens (3 mm from both distal and proximal ends of the normal tibia) were taken from the surgery side of four rabbits in each group, and fixed in 10% formalin (Yilin Chemical Plant, Nanjing, China). The specimens were decalcified, soaked in gelatin, embedded in methyl methacrylate (Yilin Chemical Plant, Nanjing, China), and serially sectioned into 5-μm slices using a freezing microtome (RM 2135 Microtome, Leica, Germany); the slices were stained with hematoxylin and eosin (automated tissue stainer, ZMN-2802) and examined with ×10 optical microscopy (Olympus, Tokyo, Japan) for histological changes during the bone graft repairing process. The images of slices were analyzed to calculate the new bone quantity using Mias Sharpvision image analytical system. When the specimen section was visualized clearly in the field, the image scale was calibrated, and the total bone tissue areas, including the surrounding new bone callus and the new bone callus area only, were measured in slices from different time points in both groups. The formula for calculation of new bone quantity was as follows: new bone quantity (%) = new bone area (mm^2^) / total sectional area of bone (mm^2^) × 100%.

### BGP measurement

Two, 4, 8, and 12 weeks after surgery, 4 ml blood was drawn from each of six random rabbits from each group, and centrifuged after coagulation at 2000 rpm; the serum was isolated. Serum bone Gla protein (BGP) level was measured using a radioimmunoassay kit.

### Biomechanical assessment

A three-point bending test was performed using a biomechanical universal tester (SLW-10) for four random rabbits from each group, 12 weeks after surgery. The periosteum and soft tissue were removed from the tibial specimen, which was fixed horizontally at both ends to the two holders and centered on the graft segment with a span length of 40 mm. A load was uniformly applied to the midpoint of the graft at a constant rate of 0.5 mm/min until fracture; data were recorded dynamically by computer, and the relationships between bending stress, compressive strength, and displacement were plotted. The mean values of maximum compressive force and compressive strength for the two groups were compared.

### Statistical analysis

All the data are presented as mean ± standard deviation (SD). Variance analysis and F-tests for results were performed using SPSS11.5 statistical software (SPSS Inc., Chicago, IL, USA). *P* < 0.05 was considered statistically significant.

## Results

### X-ray examination

**Fig. 2:**
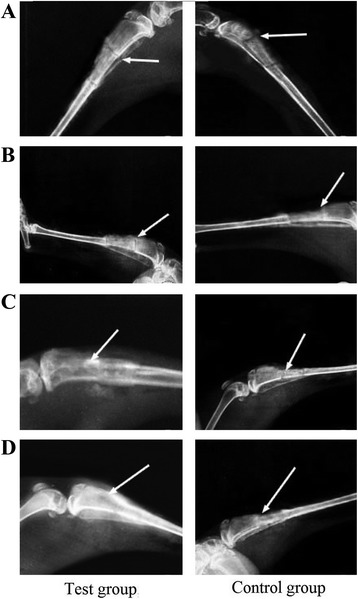
X-ray examination after surgery. **a** 4 weeks after surgery. **b** 8 weeks after surgery. **c** 12 weeks after surgery. **d** 16 weeks after surgery

In the test group, 4 weeks after surgery, the fracture line could still be observed. There was low-density flake-like bone callus shadow and partial bone formation. After 8 weeks, the fracture line turned vague and more bone callus was observed. The medullary cavity started to recanalize. After 12 weeks, the fracture line practically disappeared, the surrounding bone callus absorption started, the medullary cavity recanalized, and the bone fracture healed. After 16 weeks, the bone continuity between host bone and graft bone recovered, good shaping annular bone callus was observed surrounded implanted bone, new cortex generated, and the medullary cavity enlarged (Fig. 2).

In the control group, after 4 weeks, the fracture line was clear. There was little dotted bone callus shadow. After 8 weeks, the fracture line turned slightly vague and bone callus was observed to move from the host bone to the graft bone. After 12 weeks, more bone callus was observed around the host and the graft bone. Part of the defect area had been repaired, but the fracture line still existed. After 16 weeks, the fracture line practically disappeared. Medium amount of bone formation was observed. The medullary cavity tended to recanalize (Fig. 2).

Lane-Sandhu Scoring results showed that the scores for the test group after 4, 8, 12, and 16 weeks from surgery were statistically significantly higher than those of the control group, respectively (*P* < 0.05, Table 1).

**Table 1:** X-ray scoring results after graft surgery (mean ± SE)

Time after surgery (week)	Test group	Control group	*F* value	*P*
4	3.74 ± 0.50	2.96 ± 0.61	5.88	<0.05
8	7.16 ± 0.56	5.83 ± 0.82	8.16	<0.05
12	9.90 ± 0.62	7.72 ± 0.46	37.55	<0.05
16	11.66 ± 0.08	10.25 ± 0.35	91.55	<0.05

### New bone quantity assessment

**Fig. 3:**
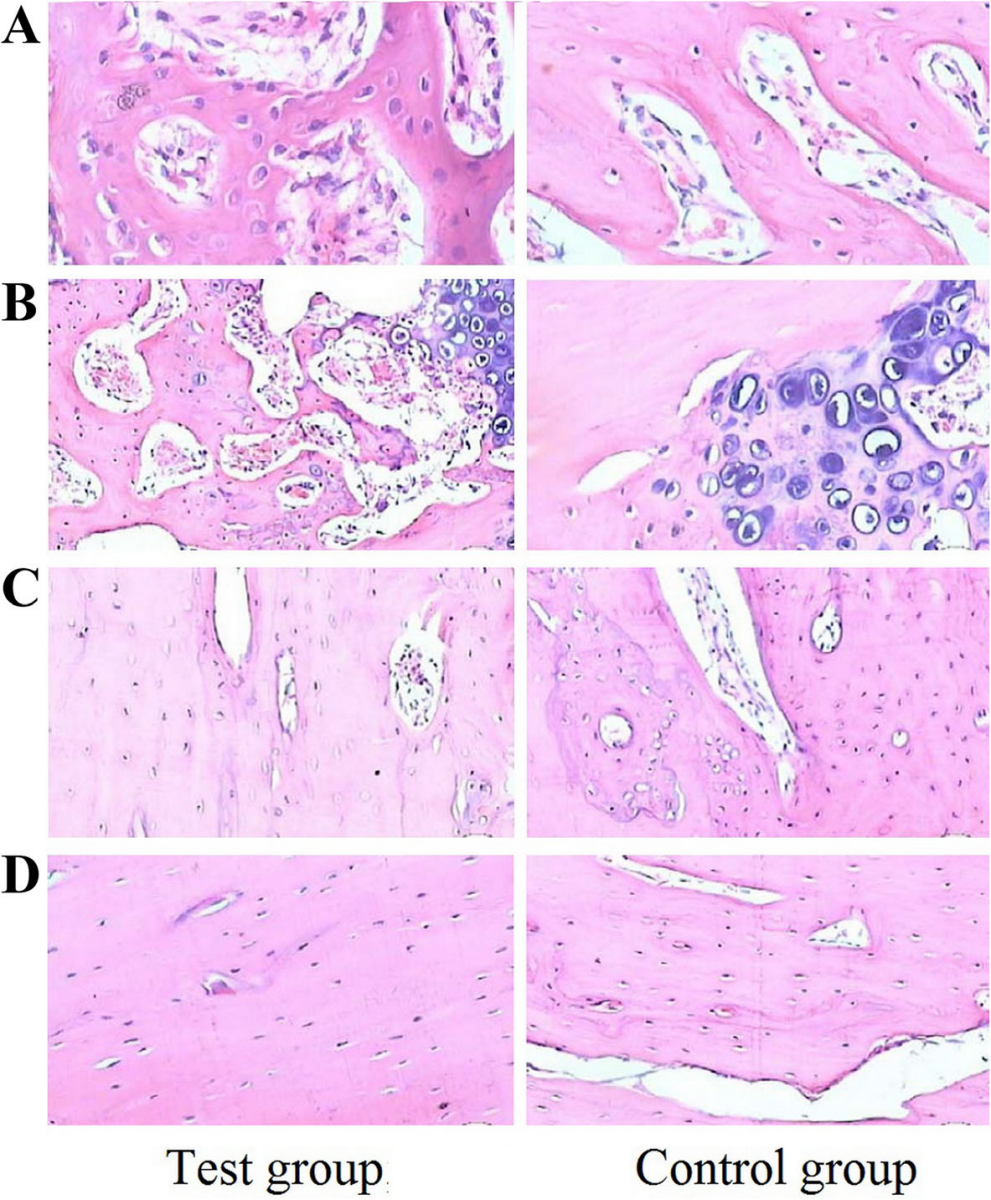
Histological slices (HE ×100). **a** 2 weeks after surgery. **b** 4 weeks after surgery. **c** 8 weeks after surgery. **d** 12 weeks after surgery

New bone quantity gradually increased in both groups after transplantation until the eighth week, and gradually decreased thereafter, but was still significantly higher after 12 weeks than after 2 or 4 weeks. Bone quantity was greater in the test group than in the control group at all time points (Fig. 3), and the difference was statistically significant (*P* < 0.05, Table 2).

**Table 2:** Change of new bone quantity after surgery (mean ± S%)

Time after surgery (week)	Test group	Control group	*F* value	*P*
2	34.13 ± 4.96	24.62 ± 3.84	10.87	<0.05
4	47.41 ± 6.04	35.12 ± 3.65	15.27	<0.05
8	73.85 ± 2.30	63.23 ± 4.18	25.57	<0.05
12	67.82 ± 5.17	59.33 ± 1.58	12.44	<0.05

### Serum BGP measurement

Serum BGP level gradually increased in both groups after transplantation until the fourth week, and gradually decreased thereafter; the difference between the two groups was further diminished, but the BGP level was statistically higher in the test group than the control group at all time points (*P* < 0.05, Table 3).

**Table 3:** Change of Serum BGP after surgery (mean ± S μg/l)

Time after surgery (week)	Test group	Control group	*F* value	*P*
2	5.75 ± 0.45	4.32 ± 0.14	60.15	<0.05
4	6.45 ± 0.30	4.46 ± 0.62	129.66	<0.05
8	5.56 ± 0.52	4.50 ± 0.79	15.32	<0.05
12	4.65 ± 0.54	4.33 ± 0.66	2.85	<0.05

### Biomechanical assessment

Twelve weeks after surgery, the maximum compressive force and compressive strength values for the test group were significantly higher than those for the control group (*P* < 0.05, Table 4).

**Table 4:** Comparison of three-point bending test results at 12 weeks after surgery (mean ± S, *n* = 5)

Group	Maximum compressive force (N)	Compressive strength (Mpa)
Test group	230.4 ± 13.4	44.3 ± 5.4
Control group	186.9 ± 22.3	34.5 ± 3.1

## Discussion

Large segmental defects are often caused by trauma, tumor, and infection and are difficult to reconstruct, especially in weight-bearing bones, which require good biomechanical properties of grafted bone [12]. In this study, we explored the effect of fascial flap-wrapped allogeneic bone on repair of large segmental tibial defects in rabbits. This could provide a theoretical basis for treatment of large segmental defects in weight-bearing bones. Results showed that fascial flap-wrapped allogeneic bone is superior to allogeneic bone alone in the treatment of large segmental bone defects; this was confirmed using postoperative X-ray examination, new bone quantity assessment, serum BGP levels, and biomechanical testing.

The treatment of bone defects has mainly relied on bone grafting using autogenous, allogeneic, xenogeneic, and synthetic bones. Autogenous bone grafting has natural advantages [13], as well as the following disadvantages: limited bone availability, more incisions, prolonged anesthesia and surgery, increased blood loss and infection incidence, damage to normal structures at the donor site, and postoperative pain and scarring at the donor site [14]. The reported incidence of donor site complications after autogenous iliac crest bone grafting is up to 30% [15]. Immunological rejection after allogeneic bone grafting can affect healing [16]. Allogeneic bones are important for reconstruction of bone defects due to good availability and histocompatibility, as well as the similarity of the healing process to that in autogenous bones, thus preventing multiple complications following autogenous bone grafting [17].

Allogeneic bones meet three requirements for bone formation [18]. (i) Sufficient number of bone-generating cells. It is thought that naive connective tissue cells in the graft bed can transform into bone-generating cells, and subsequently into osteoblasts and bone cells with stimulation. (ii) Good blood supply. Bone formation is faster with a good blood supply surrounding grafted allogeneic bone. (iii) Appropriate trigger factors. Bone morphogenetic protein (BMP) in allogeneic bones can promote osteogenesis. Herrera et al. [19] identified the roles of allogeneic bone grafts as follows: (i) structural support to maintain fracture reduction and (ii) osteoconduction substrate with specific osteoinductive effects. Currently, allogeneic bones are widely used as clinical bone grafting materials.

Gillies et al. [20] and Delloye et al. [21] recognized the importance of the deep fascia in skin blood flow. Heiple et al. [22] demonstrated the abundant vascular network in the deep fascia by vascular dye perfusion in fresh cadavers and animal experiments. Thus, use of pedicled fascial tissue or wrapping of ischemic tissue with greater omentum to establish blood circulation is feasible for allogeneic bone revascularization. Vuppalapati et al. [23] reported that vascularized fascial flap-wrapped allogeneic bones are superior to allografts following vascular bundle implantation for bone defect reconstruction. Wilkins et al. [24] established a model of pedicled fasciocutaneous flaps based on unknown vessels in the rabbit forearm, and used a flap-wrapped, composite cell-biomaterial to repair 1.5-cm mid-radius bone defects in rabbits. They found that revascularization is significantly faster in tissue-engineered bone wrapped with fascia flaps than in bone without fascia flaps after bone defect reconstruction, indicating that flap wrapping can facilitate vascularization in the initial bone formation process by massively transporting host-targeting mesenchymal cells to grafted bones through early stage, fast blood capillary proliferation.

Thus, the treatment of large segmental bone defects often uses single bone grafts and vascularized bone tissue transplantation. The combined application of allogeneic bone and surrounding fascial tissue is reported less often. In this study, vascularized fascial flap-wrapped allogeneic bone was used for repair of large segmental bone defects. The results were superior to those using bone grafts alone. This has provided a basis for the repair of segmental weight-bearing bone defects.

## Conclusions

Fascial flap-wrapped allogeneic bone to repair of large segmental tibial defects has the advantages of allogeneic bone transplantation and the function of fascial-flap vascularization, with synergistic and additive effects on osteoblasts. The method can shorten the time of fracture healing, and is ideal for the repair of large segmental bone defects.

## Abbreviations

BGP: Serum bone Gla protein
BMP: Bone morphogenetic protein
